# Does health literacy affect the uptake of annual physical check-ups?: Results from the 2017 US health information national trends survey

**DOI:** 10.1186/s13690-021-00556-w

**Published:** 2021-03-18

**Authors:** Hee Yun Lee, Sooyoung Kim, Jessica Neese, Mi Hwa Lee

**Affiliations:** 1 School of Social Work, University of Alabama, AL Tuscaloosa, USA; 2 Dept. of Aging and Social Work, College of Nursing, Catholic University of Pusan, Busan, South Korea; 3 School of Social Work, East Carolina University, Greenville, North Carolina USA

**Keywords:** Health literacy, Physical check‐ups, Older adults, Young adults, Andersen behavioral model

## Abstract

**Background:**

Little is known about how health literacy is linked to physical check-ups. This study aimed to examine the levels of physical check-ups (self-reported check-ups within the last year) by age group (those aged 18–59 years and those aged = ≥ 60 years) and the role of health literacy regarding physical check-ups in the United States.

**Methods:**

Data for the study were obtained from the 2017 Health Information National Trends Survey. The original sample included 3,285 respondents, but only 3,146 surveys were used for this study. Andersen’s Behavioral Model of Health Services Use guided this study, and a binomial logistic regression model was conducted using Stata 12.0 software package.

**Results:**

While 82.0 % of the older group had an annual check-up, 67.3 % of the younger group had one. Both groups had similar ratios for health literacy-related item reporting. Study results show that annual check-up was positively associated with confidence in getting health information, having health insurance, and having a primary doctor for both age groups. However, getting a regular check-up was negatively associated with frustration while searching for information among the younger group. In comparison, it was positively associated with difficulty understanding information for the older group.

**Conclusions:**

To increase annual physical check-ups, health literacy-related interventions should be developed and address the barriers most associated with health check-ups. One way of addressing this barrier is to improve communication from healthcare professionals to consumers through the use of easy-to-understand explanations appropriate for the consumer.

## Background

Routine physical check-ups offer multiple health benefits that can lead to a longer, healthier life. Regular check-ups, defined as a routine test primary care provider performs to check overall health, are used to assess individuals’ general health and prevent future illnesses [1]. Check-ups give health care providers an opportunity to get to know their patients better [2], allow for early detection of health problems in the beginning or early stages and offer better treatment chances [3, 4], and can be cost-saving [4]. Despite the benefits of regular check-ups, some argue there is no clear evidence to support the need for physical check-ups [5], while others believe that annual check-ups increase diagnoses and medications but does not affect ways to decrease morbidity and mortality from diseases such as cardiovascular issues and cancer [2]. Contrary to critics’ arguments, physical check ups are still needed to continue identifying and detecting diseases and other health issues that individuals experience early [6]. By being at the forefront of these diseases and issues, physicians can provide individuals with the appropriate services or referal as needed and reduce patients’ concerns [1].

Previous studies have reported various barriers and facilitators associated with regular check-ups, including socio-demographic characteristics (e.g., age, gender, income) [7–9], accessibility to health care services (e.g., health insurance, primary doctor, and living area) [10–13], personal cancer history [14, 15], and family cancer history [16]. For instance, individuals in rural areas were less likely to have physical check-ups because obtaining a primary care doctor was difficult as physicians are typically in cities and more affluent suburbs, and having low income was associated with not seeing a doctor for check-ups because of cost [17]. On the other hand, young adults between the ages of 18–26 with a usual source of care were more likely to utilize physical check-ups [18], and having health insurance increased the likelihood of routine check-ups [13]. Another facilitating factor includes having a history of physical check-ups. Labeit and colleagues [4] concluded that individuals who visited a general physician in the past year were more likely to make an appointment for the coming year, suggesting that once the behavior of annual check-ups is initiated, the behavior will continue. Interestingly, individuals with a history of cancer were more likely to utilize check-ups than those without a history [14, 15], but having a family history of cancer did not increase one’s routine check-ups use [16].

In addition to the literature supporting factors associated with physical check-ups, health literacy could be another critical factor to explain an individual’s physical check-ups [19, 20]. For example, people with limited health literacy tend to have cancer screenings and immunizations less frequently [21]. However, little is known about how health literacy is linked to physical check-ups. To our best knowledge, this is the first study to investigate the contribution of health literacy to the uptake of physical check-ups.

Moreover, previous studies showed that age was a significant factor that was relevant for the utilization of check-ups [22, 23]. Other studies also reported that over 60 years old age showed significant health problems when compared to different age groups [24–26]. It will be very crucial to investigate the differences in uptakes of check-ups by age groups.

Hence, this study aimed to examine the levels of physical check-up uptake and factors associated with physical check-ups with specific attention to health literacy’s role on physical check-up uptake in two age groups. In our study, health literacy was defined as an individuals’ ability to obtain, process, and understand basic health information to make responsible decisions regarding their health [27, 28].

### Conceptual framework

The Andersen’s Behavioral Model of Health Services Use [29] guided this study. The Andersen’s Behavioral Model has been used extensively to examine relationships between predisposing, enabling, and need factors and health service utilization [22, 23, 30, 31]. The Andersen model is commonly used in studies on various health services divisions and diseases, such as HIV, dental, and long-term care [32–34]. The model has also been used to predict variables associated with health literacy [30, 31, 35].

According to the Andersen model, individuals’ access to and use of health services are explained by three components of predisposing, enabling, and need factors [29]. The model purports that health service utilization is dependent on individuals’ propensity to use services (predisposing), their ability to access services (enabling), and their illness level (need) [22]. The model believes that three types of individual factors facilitate or impede access to and utilize health care services [23]. Predisposing factors identified in health care settings include sociodemographic determinants such as age, gender, marital status, socioeconomic status (SES), and family status [22, 23, 30]. Enabling factors include education, primary care physicians, health insurance coverage, availability of health services, and social support [22, 23, 30]. Need factors include medical conditions and depression/anxiety [22].

**Fig. 1:**
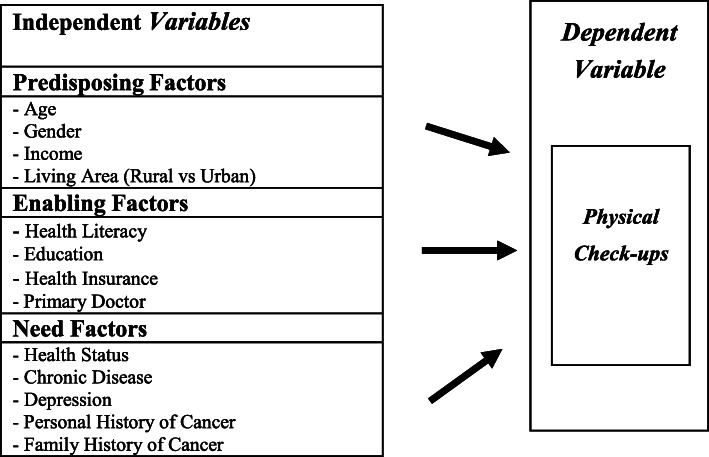
Conceptual Framework of uptake of physical check-ups in the United States

As predisposing factors according to the Andersen model, we consider age, gender, income, and living area which has individual’s social-cultural characteristics. As enabling factors according to the Andersen model, health literacy, education, health insurance, and primary doctor, which reflect conditions making healthcare available to individuals, were considered. Need factors include an individual’s beliefs on their health and access to services such as self-reported health and the number of diseases (e.g., chronic disease, depression, cancer, etc.) and personal and family history of cancer. Figure 1shows the conceptual model of this study.

## Methods

### Data and sample

The current study’s data is derived from the US-based 2017 Health Information National Trends Survey (HINTS). The National Cancer Institute administers the HINTS program to assess the American’s health information use and health behavior. Briefly, a single-mode mail survey was generated and dispersed from January to May 2017. HINTS was administered in two languages, English and Spanish. The original sample included 3,285 respondents, but our study sample included 3,146. To achieve the present study’s objectives, we excluded those aged under 18 years (N = 139). The sample was categorized into two subgroups: those aged 18–59 years and those aged = ≥ 60 years. Since World Health Organization (WHO) suggested that most developed countries characterize old age starting and 60 years and above [36].

Overall, the sample consisted of 1,681 respondents aged 18–59 years and 1,465 respondents aged = ≥ 60 years.

### Measures

#### Dependent variables

The dependent variable measured respondents’ self-reported check-ups within the last year (1 = Yes, 0 = No). The routine check-ups meant a general physical exam. It did not include an exam for a specific injury, illness, or condition.

#### Independent Variables

Three sets of independent variables were included, representing the Andersen model’s predisposing, enabling, and need factors.

1. Predisposing factors: Four predisposing factors included age, gender (male = 0; female = 1), income, and living area (rural = 0; urban = 1). Income was measured with nine categories (1 = $0–9,999; 2 = $10,000–14,999; 3 = $15,000–19,999; 4 = $20,000–34,999; 5 = $35,000–49,999; 6 = $50,000–74,999; 7 = $75,000–99,999; 8 = $100,000–199,999; 9 =≥$200,000). But collapsed into two groups (less than $74,999 = 0; $75,000 or more = 1) only for descriptive statistics. Living area was measured as rural or urban and the areas were defined by the 2013 Rural-Urban Continuum Codes.
2. Enabling Factors. Four enabling factors included health literacy, education, health insurance, and primary doctor. Health literacy was measured using five items: (1) it took great effort to get the information you need, (2) you felt frustrated during your search for information, (3) you were concerned about the quality of the information you found, (4) the information you found was hard to understand, and (5) confidence in getting health information. The first four items are based on a 4-point scale ranging from *strongly disagree* (1), *disagree* (2), *agree* (3), and to *strongly agree* (4). For analysis, all items were dichotomized (0 = disagree; 1 = agree). The last health literacy item (confidence in getting health information) was measured on a 5-point scale from *not confident at all* (1), *a little confident* (2), *somewhat confident* (3), *very confident* (4), *and* to *completely confident* (5). For descriptive analysis, it was dichotomized as not very confident (0) or very confident (1). The internal consistency (Cronbach’s α) of the five items in the full sample was 0.911. Education was measured by seven categories (1 = *less than eight years*; 2 = *eight through 11 years*; 3 = *12 years or completed high school*; 4 = *post-high school training other than college*; 5 = *some college*; 6 = *college graduate*; 7 = *postgraduate*) and the level of education was dichotomized as under some college (0) or some college and above (1) for descriptive analysis. The two variables of health insurance and primary doctors were measured using a *yes* (1) or *no* (0) question.
3. Need Factors. Five need factors consisted of self-rated health status, chronic disease, depression, personal history of cancer, and family history of cancer. Health status was measured by a single question on a five-scale from *poor* (1) to *excellent* (5). Chronic disease was measured using five items: (1) Diabetes or high blood sugar (2) High blood pressure or hypertension (3) A heart condition such as heart attack, angina, or congestive heart failure (4) Chronic lung disease, asthma, emphysema, or chronic bronchitis (5) Arthritis or rheumatism. These items were measured using a *yes* (1) or *no* (0) question and the total score was computed by summing five items. Depression was measured using the four-item Patient Health Questionnaire (PHQ-4) (i.e., little interest or pleasure in doing things; feeling down, depressed, or hopeless; feeling nervous, anxious, or on edge; not being able to stop or control worrying). All the items were on a four- scale from *not at all* (1) to *nearly every day* (4), with a higher score indicating a high level of depression. The total score was computed by summing four individual items. The sum of each variable ranged from 4 to 16. The internal consistency (Cronbach’s α) of the four items in the full sample was 0.942. Two questions of cancer history were measured if the respondent has cancer (*yes* = 1; *no* = 0) and if their family has cancer (*yes* = 1; *no* = 0).

### Data analysis

Our analytic process involved two steps. In step one, descriptive statistics were calculated to generate frequencies and proportions for sociodemographic characteristics of the sample and the health literacy and check-up variables. These statistics were calculated by younger and older age groups. Differences between younger and older respondents were examined using χ2 tests with an α level of 0.05. In the second step, to examine health literacy’s role on a physical check-up uptake among study samples, we used a binomial logistic regression model with adjustments for predisposing factors, enabling factors, and need factors. All analyses applied jackknife weighting procedures provided by HINTS for analysis of the intricate survey design and were conducted using survey procedures in Stata version 12.0 [37]. This way allowed us to produce a valid variance estimation that eventually led us to produce unbiased estimates.

## Results

### Sociodemographic Characteristics

Table 1 presents the sociodemographic characteristics by age group. Of the 1,681 respondents in the 18–59 age group, 67.3 % had an annual check-up within the past year and 82.0 % of the 1,465 respondents in the 60 years or older group. The average age was 44 years (SD = 10.737) in the younger group and 70 years (SD = 7.999) in the older group. There were relatively more females than males in both age groups. About 56.41 % of the younger group and 72.42 % of the older group members earned <$75,000 per year. Most (88.58 % and 84.37 %, respectively) participants lived in an urban area. Two-fifths of the younger group completed some college or higher, and nearly half (41.18 %) of the older group had a high school diploma or less. The majority in both groups had health insurance. More than a third in the younger group and most (81 %) of the older group had a primary doctor, and 86.1 % of the younger and 77.79 % of the older group reported their health as more than good. The average number of chronic diseases was higher in the older group (1.613, SD = 1.178) than the younger group (0.763, SD = 1.009). The average depression level was higher in the younger group (6.069, SD = 2.923) than the older group (5.747, SD = 2.617). Only 7.74 % had ever had cancer in the younger group and 24.16 % in the older group.

**Table 1:** Estimates for Sociodemographic and check-ups by Age Groups in the United States, the 2017 US Health Information National Trends Survey

	Frequency (Mean/Number)	Check-ups			Frequency (Mean/Number)	Check-ups		
		**Age between 18 and 59**			**Age 60 and Over**			
**Variables**	**Mean** **(S.D.) /Total(%)**	**No**	**Yes**	$${x}^{2}$$	**Mean** **(S.D.) /Total(%)**	**No**	**Yes**	$${\varvec{x}}^{2}$$
		**%**	**%**			**%**	**%**	
**Dependent Variable**								
Check-up	0.673(0.469)	**32.75**	**67.25**		0.820(384)	**18.02**	**81.98**	
**Predisposing factor**								
Age	44 (10.737)				70(7.999)			
Gender								
Male	665(39.82)	36.84	63.16	8.3826**	598(41.61)	19.40	80.60	1.4497
Female	1,005(60.18)	30.05	69.95		839(58.39)	16.92	83.08	
Income								
$0 to $74,999	898(56.41)	34.97	65.03	4.697*	927(72.42)	18.12	81.88	0.904
$75,000 or more	694(43.59)	29.83	70.17		353(27.58)	15.86	84.14	
Living Area								
Urban	1,489(88.58)	32.84	67.16	0.089	1,236(84.37)	18.28	81.72	0.374
Rural	192(11.42)	31.77	68.23		229(15.63)	16.59	83.41	
**Enabling Factors**								
A lot of Effort to get the information								
Agree	436(32.06)	37.84	62.16	8.841**	430(38.84)	16.74	83.26	0.011
Disagree	924(67.94)	29.76	70.24		677(61.16)	16.99	83.01	
Frustrated during search for the information								
Agree	422(31.05)	40.05	59.95	16.449***	360(33.30)	18.61	81.39	1.214
Disagree	937(68.95)	28.92	71.08		721(66.70)	15.95	84.05	
Concerned about the quality of the information								
Agree	731(53.83)	35.02	64.98	4.962*	485(44.58)	16.49	83.51	0.066
Disagree	627(46.17)	29.35	70.65		603(55.42)	17.08	82.92	
Information you found was hard to understand								
Agree	288(21.25)	34.03	65.97	0.485	312(28.55)	16.99	83.01	0.007
Disagree	1,067(78.75)	31.87	68.13		781(71.45)	16.77	83.23	
Confident get Health information								
Very confident	1,046(63.70)	30.21	69.79	8.142**	842(59.38)	16.03	83.97	5.343*
Not very confident	596(36.30)	37.08	62.92		576(40.62)	20.83	79.17	
Education								
**High school deploma or less**	433(25.88)	36.26	63.74	3.370	598(41.18)	17.56	82.44	0.211
**Completed some college or higher**	1,240(74.12)	31.45	68.55		854(58.82)	18.50	81.50	
Health Insurance								
Yes	1,560(93.41)	30.58	69.42	35.753***	1,411(97.51)	16.80	83.20	11.950***
No	110(6.59)	58.18	41.82		36(2.49)	38.89	61.11	
Primary doctor								
Yes	1,072(64.19)	22.67	77.33	129.341***	1,173(81.01)	13.55	86.45	57.508***
No	598(35.81)	49.83	50.17		275(18.99)	32.73	67.27	
**Need factors**								
Health Status								
More than Good	1,437(86.10)	33.54	66.46	2.767	1,131(77.79)	17.95	82.05	0.017
Less than Fair	232(13.90)	28.02	71.98		323(22.21)	18.27	81.73	
Chronic Diseases	0.763(1.009)				1.613(1.178)			
Depression	6.069(2.923)				5.747(2.617)			
Personal History of Cancer								
Yes	130(7.74)	33.23	66.77	2.164	353(24.16)	16.71	83.29	0.578
No	1,550(92.26)	26.92	73.08		1,108(75.84)	18.50	81.50	
Family History of Cancer								
Yes	1,153(73.49)	31.74	68.26	0.799	1,069(77.02)	17.40	82.60	0.499
No	416(26.51)	34.13	65.87		319(22.98)	19.12	80.88	

Note: * ***p*** < .05; ^**^p < .01, ^***^p < .001

Second, the younger and older groups had similar ratios for health literacy-related item reporting. Nearly a third put forth a lot of effort to get information (32.06 %, 38.84 %) and felt frustrated during searches for information (31.05 %, 33.3 %). About a fourth (21.25 %, 28.55 %) reported that understanding the information they had found was difficult, yet almost 60 % of respondents in both groups (63.7 %, 59.38 %) reported that they felt confident getting health information.

Lastly, as can be seen by the cross-tabulated frequencies in Table 1, there were significant relationships between health insurance (χ^2^ = 35.753, *p* < .000, χ^2^ = 11.950, *p* < .000), primary doctor (χ^2^ = 129.341, *p* < .000, χ^2^ = 57.508, *p* < .000), confidence in getting health information (χ^2^ = 8.142, *p* < .01, χ^2^ = 5.343, *p* < .5) and uptake of check-ups for both age groups and gender (χ^2^ = 8.383, *p* < .01), income (χ^2^ = 4.697, *p* < .05), great effort required to get information (χ^2^ = 8.841, *p* < .05), frustration while searching for information (χ^2^ = 16.449, *p* < .000), concerns about the quality of retrieved information (χ^2^ = 4.962, *p* < .05) and uptake of check-ups among the younger group.

### Factors associated with Physical Check-ups

Estimates from the binominal logistic regression model presented in Table 2 show that annual check-up associated positively with health insurance (OR = 2.576, 95 % CI = 1.612–4.118, OR = 2.341, 95 % CI = 1.024–5.352), primary doctor (OR = 2.636, 95 % CI = 2.065–3.363, OR = 2.361, 95 % CI = 1.658–3.363) and chronic disease (OR = 1.435, 95 % CI = 1.235–1.668, OR = 1.438, 95 % CI = 1.228–1.685) for both age groups. However, the dependent variable was negatively associated with frustration while searching for information (OR = 0.758, 95 % CI = 0.617–0.933) and positively associated with age (OR = 1.016, 95 % CI = 1.004–1.029), gender (OR = 1.377, 95 % CI = 1.084–1.751) and confidence getting health information (OR = 1.154, 95 % CI = 1.000-1.332) among the younger group and positively associated with the term information you found was hard to understand (OR = 1.428, 95 % CI = 1.069–1.909) for the older group.

**Table 2:** Logistic Regression on Physical Check-Ups by Age Groups in the United States, the 2017 US Health Information National Trends Survey

Variables		Age between 18 and 59 (Model 1)		Age over 60 (Model 2)	
		**OR**	**95% CI**	**OR**	**95% CI**
**Predisposing factors**	Age	1.016**	1.004, 1.029	1.008	0.988, 1.029
	Gender(ref=male)	1.377**	1.084, 1.751	1.242	0.912, 1.691
	Income	0.972	0.915, 1.032	1.061	0.976, 1.154
	Urban(ref=rural)	1.092	0.755, 1.578	0.782	0.504, 1.214
**Enabling factors**	A lot of Effort to get the information (HL)	0.945	0.768, 1.162	1.009	0.769, 1.323
	Frustrated during search for the information (HL)	0.758**	0.617, 0.933	0.835	0.629, 1.108
	Concerned about the quality of the information(HL)	1.029	0.878, 1.205	1.017	0.809, 1.278
	Information you found was hard to understand(HL)	1.225	0.997, 1.504	1.428*	1.069, 1.909
	Confident Get Health Information (HL)	1.154*	1.000, 1.332	1.183	0.985, 1.420
	Education	1.071	0.981, 1.170	0.980	0.882, 1.089
	Health Insurance	2.576***	1.612, 4.118	2.341*	1.024, 5.352
	Primary doctor	2.636***	2.065, 3.363	2.361***	1.658, 3.363
**Need factors**	Health Status	1.017	0.879, 1.177	0.992	0.826, 1.193
	Chronic Disease	1.435***	1.235, 1.668	1.438***	1.228, 1.685
	Depression	0.974	0.933, 1.018	0.947	0.889, 1.010
	Personal history of cancer	0.857	0.542, 1.353	0.999	0.694, 1.439
	Family history of cancer	0.963	0.741, 1.251	0.787	0.543, 1.140
Number of observations		1,465		1,681	
Pseudo R^2^		**0.104**		**0.07**	
Log Likelihood Rate Test		**199.07**		**84.01**	

Note: * *p*<.05; ^**^p<.01, ^***^p<.001

## Discussion

Guided by Andersen’s Behavioral Model, the current study examined the levels of physical check-ups and factors associated with physical check-ups with a specific focus on the role of health literacy in the uptake of physical check-ups in two age groups. Of the older group participants, 82 % reported an annual check-up within the last year, while only 67.3 % of the younger group reported the same. Our findings are consistent with previous studies that the older group received more physical check-ups than younger adults [7, 38–40]. Among the Korean participants of a similar study, 29.5 % of the older age group regularly visited the doctor, and only 8 % of the younger group reported regular visits, further proving that older adults utilize regular visits to the doctor more than younger adults [40]. It might be a rational assumption that older adults take action as the onset of adverse health issues arise rather than waiting like younger generations who are less likely to experience health issues and have a positive perception about their health.

The results from binominal logistic regression analysis indicated that two predisposing factors (age and gender) in the younger group and three enabling factors (health literacy, health insurance, and primary doctor) and one need factor (chronic disease) in both age groups were significant factors of an annual check-up. In the younger age group, older and female participants tended to get an annual check-up more than their male counterparts. Previous studies report that women visit their primary care clinic and use preventive care services more often than men [8, 41]. Such behaviors might be rooted in traditional women’s roles and responsibility in managing a family’s health [42] and men’s lack of help-seeking behavior. Men tend to feel weak and vulnerable in help-seeking situations and viewing health symptoms as minor or insignificant [43, 44].

The current study indicated that health literacy is an important enabling factor of annual check-ups [45]. Participants with higher health literacy were more likely to obtain check-ups [45] and cancer screening rates [46, 47]. This study found that three different items of health literacy predicted annual check-up in both age groups. For the younger group, feelings of frustration when searching for information negatively influenced uptake of annual check-ups; however, most older adults indicated that difficulty in understanding information was positively associated with annual check-ups. It seems that younger adults who experienced frustration while searching for information, did not want to seek medical guidance from providers receive check-ups. In contrast, difficulty understanding health information for the older age group, could potentially be the motivator in  pursuing physical check-ups to ask their health care providers for the meaning and accuracy of health information. As a first step toward promoting routine check-ups, policy interventions improving health literacy that differ by age groups are required. Through this, it can be expected to enhance the check-ups rate of both age groups.

Although both groups expressed health information challenges, whether obtaining or understanding, study results indicated that annual health check-ups were positively associated with confidence in getting health information in the younger age group. Confidence in obtaining health information can stem from having reliable sources of information via the web, social media, friends, and a primary care doctor to provide more information to the people, and knowledge allows the transition to improved regular check-up behavior. These reliable sources of information also impacted health literacy levels among participants of the 2003 National Assessment of Adult Literacy [48]. Participants with proficient levels of health literacy relied on the internet or personal contacts, such as health care professionals, to answer health-related questions, those with basic or intermediate health literacy levels relied on newspapers or magazine, and below basic health literacy, individuals gathered their healthcare information mainly from either the radio or television [48].

Other enabling factors associated with annual check-up were health insurance coverage and a primary doctor for both groups. Study participants who had health insurance and a primary doctor were more likely to get annual check-ups than those who did not have either. This finding is not surprising given that health insurance and having a primary doctor are key factors in accessing health care and utilizing preventive health care. A previous study also indicated that persons who had health insurance were more likely to obtain check-ups [49]. On the other hand, the imporatance of health literacy is significant, even for persons with neither health insurance nor a primary doctor.

Lastly, Chronic disease was the most powerful predictor of annual check-ups for both groups. The recent study also showed that persons with chronic diseases were more likely to have check-ups [49].

## Limitations

While the current study findings provide insight into the association between health literacy and physical check-up, some limitations exist. Due to the study’s design, a correlation could be difficult to identify between health literacy and physical check-up; however, longitudinal studies are needed to explore the causal relationship between health literacy and physical check-up. Second, the explanatory power of the identical model is low. This model could explain only 9.0 % and 5.25 % of the total variance between 18 and 59 years and aged over 60 years, respectively. Some other important factors may explain the variance of health check-ups among specific age groups. Third, it’s crucial to consider environmental factors and personal factors when using Andersen’s Behavioral Model. However, we only include living area (rural or urban) as an environmental factor since 2017 HINTS data does not provide related information. Another limitation worth noting is the outcome measure (physical check-up) was self-reported rather than clinically or behaviorally measured, which might have caused response biases**.**

## Conclusions

Several methods to increase annual health check-up are suggested. First, many participants in our study showed frustrations in searching for health information and difficulty understanding the meaning of professionals’ medical terminologies. Health care professionals have the responsibility to share healthcare information with their patients and the larger community by using the right communication strategies. Medical information should be translated into easy-to-understand language by healthcare professionals. Additionally, policies should recommend medical facilities or primary doctors to provide routine reminders via call/text/email regarding upcoming appointments as it may promote awareness and enhance health literacy to include health check-ups in the person’s agenda [39, 50]. Moreover, it is critical for health care professionals and policymakers to have different strategies for each age group to enhance health literacy. For a younger age group, providing easily accessible health information via the internet and cultivating the capacity to find health information would be crucial. The ability to obtain accurate medical information quickly and conveniently online may provide an opportunity for better-informed decision making. At the same time, for older age groups, providing education to improve understanding of health materials should be provided [51, 52]. Lastly, it is important to increase preventive medical service utilization, such as annual check-ups, to prevent health deterioration. The Institute of Medicine [53] states that the individual’s efforts alone have limitations in improving health literacy. Therefore, it will be necessary to understand the mutual function between the individual and the medical environment and environmental changes. Moreover, efforts to decrease the barriers in accessibility for regular health check-ups should be accompanied by bringing awareness and service to the community with notable efforts from the health care settings.

## Data Availability

Health Information National Trends Survey is a public use dataset that is available from .

## Abbreviations

HINTS: Health Information National Trends Survey

## Declarations

### Footnotes

The total number of respondents in each age group are different since we applied the jackknife weighting procedures.
